# Experimental Study on the Flexural Performance of Composite Beams with Lipped Channels

**DOI:** 10.3390/ma17246128

**Published:** 2024-12-14

**Authors:** Changyong Lee, Byungho Bae, Seunghun Kim, Taesoo Kim

**Affiliations:** 1 Graduate School of Architectural Engineering, Hanyang University Seoul, Seoul 04763, Republic of Korea; moh623@hanyang.ac.kr; 2 NRC Structure Laboratory Co., Ltd., Seoul 08503, Republic of Korea; baebho@nrckuzo.com; 3 Department of & Architectural Engineering, Hanbat National University, Daejeon 34158, Republic of Korea; kimsh@hanbat.ac.kr; 4 School of & Architecture & Architectural Engineering, Hanyang University ERICA, Ansan 15588, Republic of Korea

**Keywords:** composite beam, flexural strength, U-shaped steel section, lipped channel, trapezoid side plate

## Abstract

This study conducted experiments to investigate the flexural behavior of steel–concrete composite beams with U-shaped sections, utilizing cold-formed lipped channels as web components. To enhance both flexural and shear performance, trapezoidal plates were added to the lower sides of the composite beams. A total of ten specimens were fabricated, with variables defined according to the following criteria: presence of bottom tension reinforcement and bottom studs, thickness of the trapezoidal side plates (6 mm and 8 mm), and the welding method. Four-point bending tests were conducted, and all specimens exhibited typical flexural failure at the ultimate state. Specimens with bottom tension reinforcement, specifically those in the H5-T6 and H5-T8 series, demonstrated increases in ultimate load of 28.8% and 33.5%, respectively, compared to specimens without tension reinforcement. The use of lipped channels enabled full composite action between the concrete and the steel web components, eliminating the need for stud anchors. Additionally, it was confirmed that the plastic neutral axis, reflecting the material test strengths, was located within the concrete slab as intended. This study also compared the design flexural strengths, calculated using the yield stress distribution method from structural steel design standards such as AISC 360 and KDS 14, with the experimental flexural strengths. The comparison was used to evaluate the applicability of current design standards.

## 1. Introduction

In the current Korean construction market, the use of steel and concrete composite beams employing cold-formed U-shaped sections is increasing, particularly in high-rise apartments and mixed-use residential–commercial buildings [1,2]. A composite beam is a structural system in which the concrete slab and steel beam act as a unified body through shear connectors. The compressive force is resisted by the upper concrete slab, while the tensile force is borne by the lower steel beam. This method has been developed and applied to maximize the advantages and minimize the disadvantages of the structural characteristics inherent to both steel and concrete. Various forms of composite structures are being developed and applied in the field [3,4,5,6].

A U-shaped composite beam filled with concrete has a high level of integrity within the member itself, as the concrete is simultaneously poured into both the slab and the interior of the steel section. Since the U-shaped steel sections are pre-assembled before being brought to the site, they can replace traditional formwork, making them highly valued from both an economic and environmental standpoint. Unlike conventional composite beams, these U-shaped filled composite beams can provide a confinement effect on the concrete due to the presence of the web steel. It has been confirmed that this also contributes to an increase in deformation capacity [7,8,9,10].

**Figure 1:**
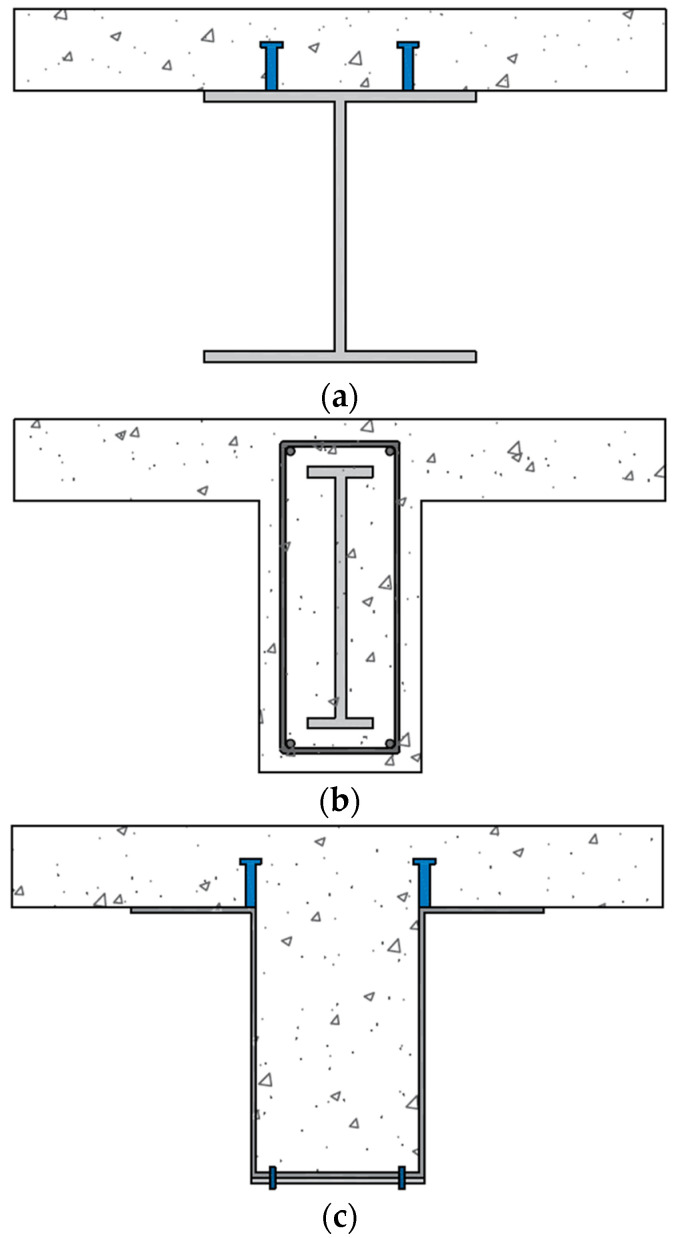
Composite beam section diagrams: (**a**) exposed; (**b**) encased; (**c**) concrete-filled.

As shown in Figure 1, traditional U-shaped composite beams are highly cost-effective and environmentally favorable because the steel components are pre-assembled with bolts before being transported to the site. However, when steel materials with different thicknesses and grades are used, multiple steel components are assembled, forming the U-shaped section through bolt or welded connections [11,12,13,14,15].

Liu et al. [11] proposed the use of U-shaped reinforcement between the concrete beam and slab to enhance both flexural and shear capacities. Stud anchors were applied to ensure full composite action between the steel and concrete. The flexural and shear performance of cold-formed U-shaped composite beams reinforced with steel trusses was evaluated. Experimental results showed that the shear connectors located at the bottom flange of the U-shaped steel contributed to shear force transfer but did not enhance the flexural capacity. After ultimate strength, concrete splitting failure occurred, leading to a reduction in shear capacity. Kim et al. [12,13,14] conducted experiments to evaluate the flexural performance of U-shaped composite beams, which involved connecting Z-shaped side plates and C-shaped bottom steel plates with high-strength bolts and then pouring concrete. The sides were made of cold-formed flat plates, and the ribs of the channel-shaped bottom steel and one flange of the Z-shaped side plates were connected with bolts. The composite effect between the steel components and the concrete was induced. The specimens using ultra-high-strength steel for the bottom plates did not exhibit sufficient load-bearing capacity and experienced a rapid decrease in strength after the ultimate strength. Kim et al. [15] evaluated the flexural performance of U-shaped composite beams by fastening Z-shaped side plates and channel sections to the bottom plate with high-strength bolts and then pouring concrete. Tests were conducted by varying parameters such as the size of the beam and slab. Experimental results showed that the specimens using high-strength steel had overestimated flexural strength and stiffness.

In existing U-shaped composite beams, it was confirmed that variations in the beam depth affect the member’s strength increase. Slip between the steel and concrete prevented the shear connectors from functioning effectively. Additionally, the flexural strength of high-strength steel was overestimated.

In this study, a U-shaped steel beam with vertically lipped channels welded to both sides of the beam web was designed, and concrete was poured inside the beam to create a composite beam. To enhance the flexural performance, trapezoidal side plates were installed on both side faces of the beam’s bottom. The flanges and lips of the channel used as web members serve as shear connectors between the internally filled concrete and the steel. The purpose of this study is to evaluate the flexural strength of composite beams with lipped channels through four-point bending tests, based on the Korea building structural design codes (KDS 41 and KDS 14) and the American Institute of Steel Construction standards (AISC 360-22) [16,17,18,19].

## 2. Experiment Plan

### 2.1. Specimen Plan

**Figure 2:**
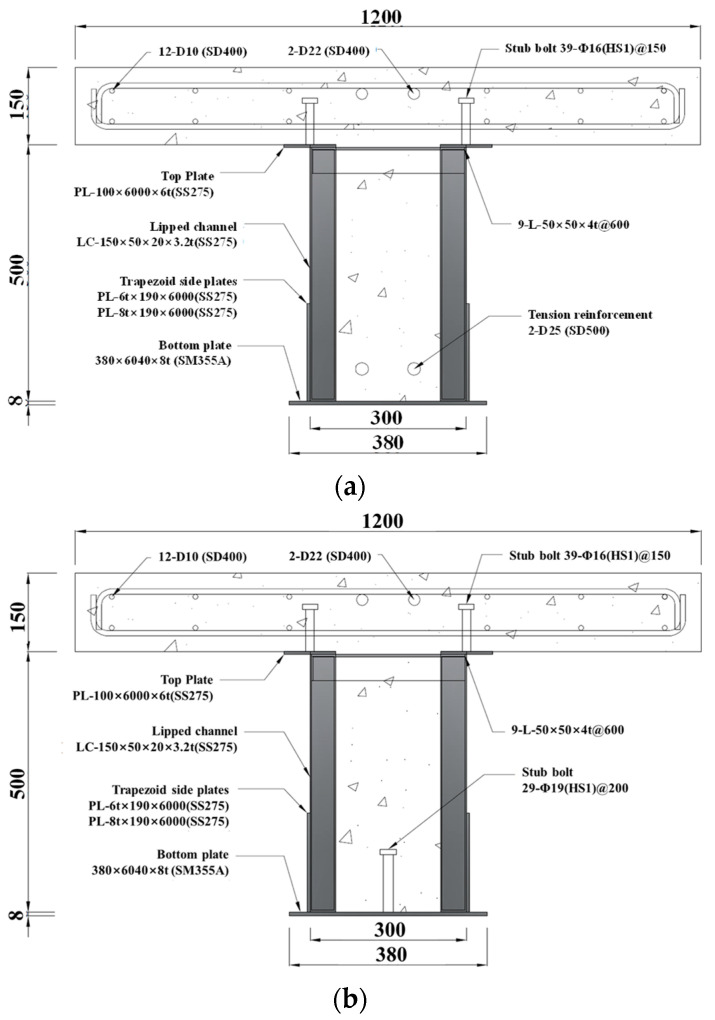

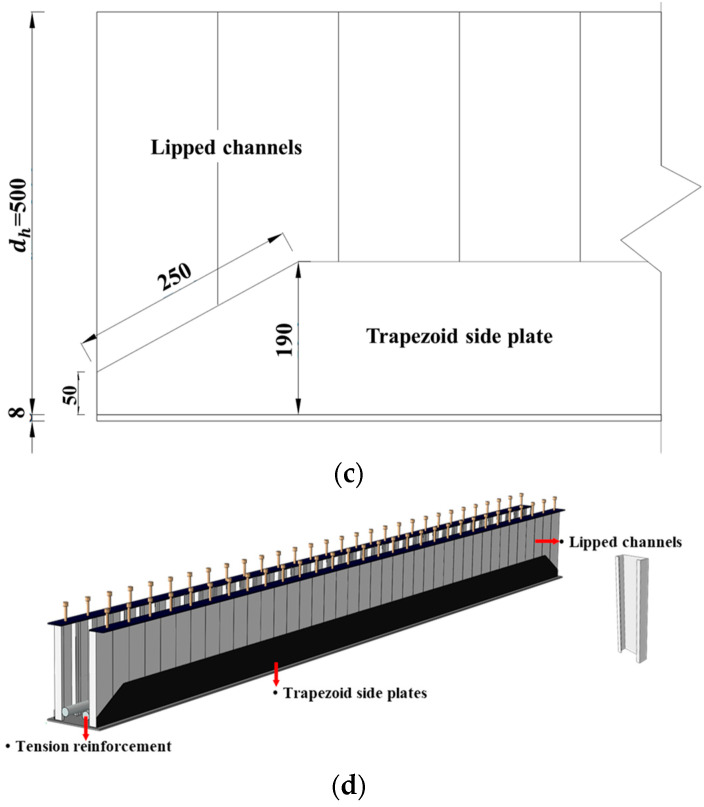
Cross-section and configuration of specimens (unit: mm). (**a**) H5-T6/T8-R/NR series; (**b**) H5-T6/T8-S/NS series; (**c**) lipped channel and trapezoid side plate; (**d**) assembly of U-shape composite beam.

Lipped channel sections improve resistance to both local and global buckling by adding lips to the channel section, as depicted in Figure 2. This enhanced structure increases the efficiency of the steel section, reducing the amount of steel used while ensuring stability. The addition of trapezoidal side reinforcement plates improves shear strength and load distribution, further enhancing flexural performance and enabling safe support of heavy loads. In the process of connecting lipped channel sections to each other and to lipped channel sections with side trapezoidal plates, excessive welding and heat-affected zones could occur. To mitigate this issue, the specimens were fabricated using intermittent welding as a variable, instead of full welding.

To evaluate the flexural performance of composite beams utilizing lipped channels and trapezoidal side plates, specimens were designed and fabricated according to the following criteria: the presence or absence of bottom tensile reinforcement (R/NR), the inclusion or exclusion of bottom studs (S/NS), the thickness of the trapezoidal side plates (6 mm/8 mm), and the welding method between the lipped channels (basic welding (B) or reinforced welding (A)). The welding methods (B/A) were also incorporated as criteria. The welding was carried out as intermittent welds applied vertically between the lipped channels at regular intervals. The number of welds connecting the trapezoidal side plates to the lipped channels was also treated as a variable. For basic welding (B), a single weld was applied at the central point where the web of the lipped channel and the trapezoidal side plate meet, with a weld size of 3 mm and a weld length of 20 mm. For reinforced welding (A), fillet welds were applied at three points along the joint line where the web of the lipped channel meets the trapezoidal side plate, using the same weld size and length as in basic welding. During the fabrication of the trapezoidal side plates, bevel welding was used to join two steel plates, as illustrated in Figure 2d.

The representative specimen names, variables, configurations, and dimensions are presented in Figure 2a–c. Unlike the previously studied high-strength bolt-assembled U-shaped composite beams [12,13,14,15], the composite beams using channel sections represent an improved U-shaped composite beam design with concrete infill. In this configuration, the channel sections and trapezoidal side reinforcement plates were welded to the bottom plate, as shown in Figure 2d. To ensure full composite action, top studs (∅16HS1) were arranged such that the compressive force of the concrete slab and the tensile force of the steel beam were both at least greater than or equal to the smaller of the two values. The trapezoidal side plates in the specimens were initially planned to use SS275 grade steel. However, during fabrication, the 6 mm plates were made of SM355A steel, while the 8 mm plates were made of SS275 steel.

The specimens were designed as T-shaped cross-section composite beams, as shown in Figure 2. The beam width was 300 mm, with a total height, including the concrete slab, planned to be 650 mm, except the bottom plate. The slab size was designed to ensure that the compressive force in the slab exceeds the tensile force in the bottom plate steel. The neutral axis of the T-shaped composite beam was planned to be located in the slab. Considering the experimental conditions and loading range for the testing machine, the concrete slab was specified with a width of 1200 mm and a thickness of 150 mm.

The bottom plate had a nominal thickness of 8 mm and was made of SM355A steel. The channel sections were made of SS275 steel with a nominal thickness of 3.2 mm. The angle sections for maintaining the spacing were all made of SS275 steel with a nominal thickness of 4 mm under uniform conditions. The bottom tensile reinforcement in the beam is D25(SD500) and was installed 50 mm above the bottom plate. Bottom studs (∅19(HS1)) were placed at 200 mm intervals inside the bottom plate. The concrete slab has a design compressive strength of 24 MPa, with top studs (∅16(HS1)) spaced at 150 mm intervals. Compressive reinforcement was D22(SD400), and temperature reinforcement and stirrups were planned to be D10(SD400).

**Figure 3:**
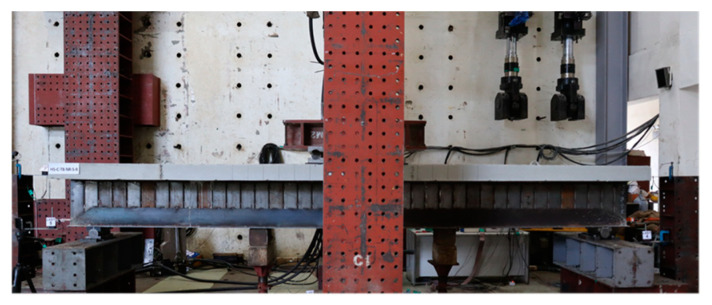
Set-up of specimens.

**Figure 4:**
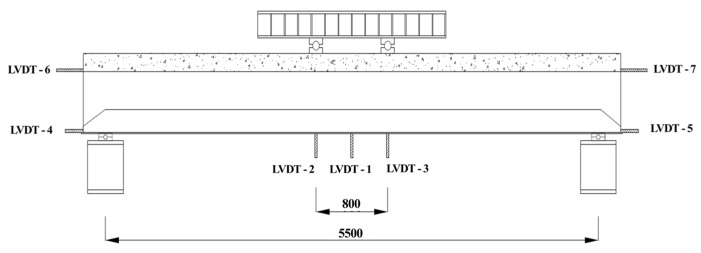
Location of LVDTs.

**Figure 5:**
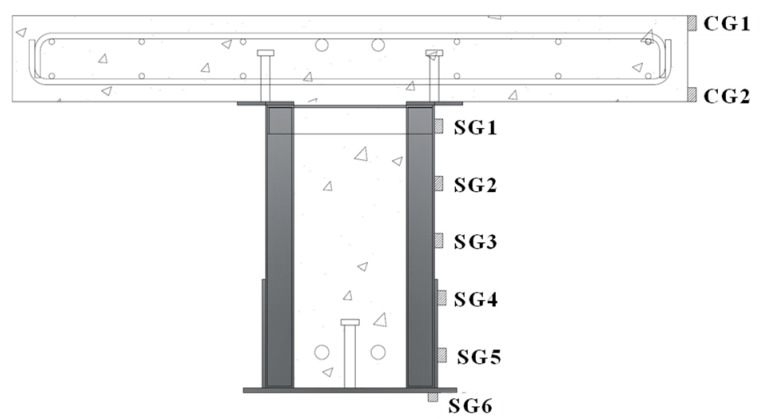
Location of strain gauge.

Figure 3 shows the set-up of the specimen. The beam was installed with simple support conditions at both ends and subjected to four-point loading. To measure vertical deflections beneath the loading points during the experiment, three displacement transducers (LVDTs 1, 2, 3) were installed on the bottom surface of the beam, as shown in Figure 4. To detect any slip between the lipped channel and the concrete, displacement transducers (LVDTs 4, 5, 6, 7) were installed on the side faces at both ends of the specimen. During the bending test, strain gauges were attached to the top surface and sides of the concrete slab, as well as to the beam web (lipped channel), trapezoidal side plates, and bottom plate, as shown in Figure 5. This set-up was used to investigate the strain change on the steel and concrete surfaces and to determine the position of the plastic neutral axis (P.N.A). Strain gauges were numbered for steel (SG) and concrete (SL/CG). The testing was conducted using a 2000 kN actuator, and the experiment was terminated when the load decreased to 80% of the maximum load after reaching the maximum load.

### 2.2. Material Test

The concrete has a design compression strength (*f_ck_*) of 24 MPa, and the concrete mix proportions are given in Table 1. Three concrete compression test specimens with a diameter of 100 mm and a height of 200 mm were prepared according to KS F 2403 [20]. These specimens were cured for 28 days and then tested for compression strength in accordance with the specifications of KS F 2405 [21]. The average compression strength of the concrete was found to be 25.0 MPa. To evaluate the material properties of the rebar, steel, and studs, tensile test specimens were prepared according to the metal materials testing standards in KS B 0801 [22]. Specimens were made for rebar (D10, D22, D25), steel plates, stud bolts (∅16, ∅19: HS1) [23], and lipped channels. Each type of specimen was prepared in triplicate. Tensile tests were conducted following the guidelines of KS B 0802 [24]. The results of the material tests are summarized in Table 2, Table 3 and Table 4.

The results of the material tests confirmed that the rebar and steel met the minimum yield strength (*f_y_*), tensile strength (*f_u_*), and elongation (*ε_elo_*) requirements specified by the Korean Standards (KS).

## 3. Experimental Results

### 3.1. Load and Displacement

**Figure 6:**
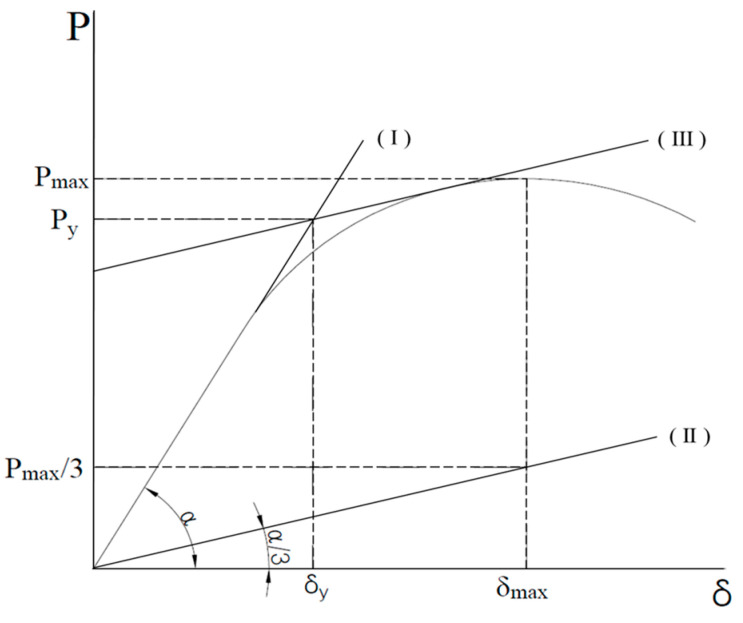
Estimation of yield load.

The yield load ($P_{y}$), maximum load ($P_{ue}$), and the displacements ($\delta_{y},\;\delta_{ue}$) at yield and maximum loads during the bending tests of the composite beams are summarized in Table 5. The yield load ($P_{y}$) of the specimens was evaluated using the 1/3 tangent method, which involves determining the yield load from the load–displacement curve by drawing a line with a slope equal to one-third of the initial slope of the curve [11,12,13,14]. As shown in Figure 6, the yield load ($P_{y}$) and yield displacement ($\delta_{y}$) were determined by the following procedure: The initial slope tangent of the load–displacement curve was used to create a line segment (I). This segment was then parallel shifted by one-third of the maximum load to form a second line segment (II). The intersection of this parallel-shifted line segment (II) with the tangent line (III) to the load–displacement curve was used to determine the yield load ($P_{y}$) and yield displacement ($\delta_{y}$). $P_{n}$ is the load calculated using the theoretical bending strength ($M_{t}$) obtained by applying the design code strength to the specimen. The maximum load of the specimen ($P_{ue}$) is the maximum load achieved during the experiment. As a result of the bending test, the ratio ($P_{ue}/P_{n}$) of the maximum load ($P_{ue}$) to the nominal load ($P_{n}$) ranged from 0.89 to 1.05, with an average of 0.97. The ratio ($P_{ue}/P_{y}$) of the maximum load to the yield load ($P_{y}$) ranged from 1.22 to 1.35, with an average of 1.30.

To evaluate the ductility of composite beams with lipped channel steel, two displacement ductility ratios were analyzed: $\delta_{ue}/\delta_{y}$, which is the ratio of the displacement at maximum load ($\delta_{ue}$) to the yield displacement ($\delta_{y}$), and $\delta_{ue75}/\delta_{y}$, which is the ratio of the displacement ($\delta_{ue75}$) at 75% of the maximum load after reaching the maximum load to the yield displacement ($\delta_{y}$). To evaluate the ductility of composite beams with lipped channel steel, two displacement ductility ratios were assessed: $\delta_{ue}/\delta_{y}$, which is the ratio of the displacement at maximum load ($\delta_{ue}$) to the yield displacement ($\delta_{y}$), and $\delta_{ue75}/\delta_{y}$, which is the ratio of the displacement at 75% of the maximum load after reaching the maximum load ($\delta_{ue75}$) to the yield displacement ($\delta_{y}$). According to this evaluation method for displacement ductility ratios, $\delta_{ue}/\delta_{y}$ and $\delta_{ue75}/\delta_{y}$, the H5-T8 series specimens showed an increase of 32.8% and 2.4%, respectively, compared to the H5-T6 series specimens in the basic welding (B) series. In the reinforcement welding (A) series, the H5-T8 series specimens exhibited increases of 5.1% and 1.2%, respectively, compared to the H5-T6 series specimens.

**Figure 7:**
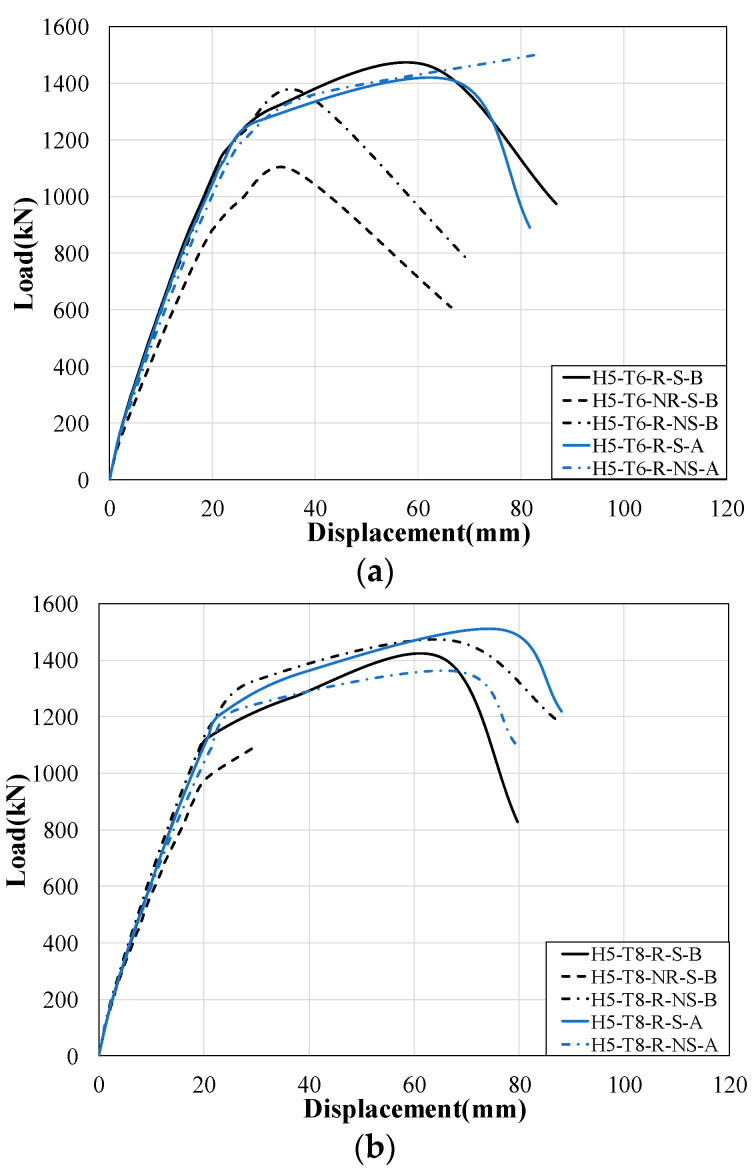
Load–displacement curves: (**a**) H5-T6 series; (**b**) H5-T8 series.

Figure 7 shows the load–displacement curves from the bending tests. Figure 7a illustrates the load–displacement curve for the H5-T6 series specimens with 6 mm trapezoidal side reinforcement plates. Based on the basic welding (B) method, the maximum load of the H5-T6-R-S-B specimen, which includes bottom tensile reinforcement, was 33.7% higher than that of the H5-T6-NR-S-B specimen, which does not include bottom tensile reinforcement. The maximum load of the H5-T6-R-S-B specimen, which includes bottom studs (∅19: HS1), was 7.2% higher than that of the H5-T6-R-NS-B specimen, which does not include bottom studs. In the specimens with reinforcement welding (A), the H5-T6-R-NS-A specimen, which does not include bottom studs, exhibited a maximum load that was 6.5% higher than that of the H5-T6-R-S-A specimen, which includes bottom studs. The H5-T6-R-S-B specimen with basic welding (B) exhibited a load capacity that was 4.1% greater than that of the H5-T6-R-S-A specimen with reinforcement welding (A).

On the other hand, the H5-T6-R-NS-B specimen, which lacks bottom studs and uses basic welding (B), showed a maximum load that was 9.1% lower than that of the H5-T6-R-NS-A specimen with reinforcement welding (A).

Figure 7b shows the load–displacement curve for the H5-T8 series specimens with 8 mm trapezoidal side plates. Based on the basic welding (B) method, the maximum load of the H5-T8-R-S-B specimen, which includes bottom tensile reinforcement, was 28.8% higher than that of the H5-T8-NR-S-B specimen, which does not include bottom tensile reinforcement. The H5-T8-R-S-B specimen, which includes bottom studs (∅19: HS1), showed a maximum load that was 9.6% lower than that of the H5-T8-R-NS-B specimen, which does not include bottom studs. The H5-T8-R-S-A specimen with bottom tensile reinforcement and reinforcement welding (A) exhibited a maximum load that was 7.2% greater than that of the H5-T8-R-S-B specimen with basic welding (B). In contrast, the H5-T8-R-NS-A specimen without bottom studs and with reinforcement welding (A) showed a maximum load that was 9.2% lower than that of the H5-T8-R-NS-B specimen with basic welding (B).

For the case with bottom tensile reinforcement, the maximum load of the H5-T6-R-S-B specimen with a nominal thickness of 6 mm for the trapezoidal side plate was 4.1% higher than that of the H5-T8-R-S-B specimen with a nominal thickness of 8 mm for the trapezoidal side plate. The maximum load of the H5-T6-NR-S-B specimen without bottom tensile reinforcement was found to be almost identical to that of the H5-T8-NR-S-B specimen. The H5-T8-R-NS-B specimen without bottom studs exhibited a 9.6% higher maximum load compared to the H5-T6-R-NS-B specimen. The H5-T8-R-S-A specimen with bottom studs and reinforcement welding (A) showed a 7.2% higher maximum load than the H5-T6-R-S-A specimen. However, in specimens without bottom studs, the maximum load of the H5-T8-R-NS-A specimen was 9.1% lower than that of the H5-T6-R-NS-A specimen.

In the initial displacement range, similar flexural behaviors were observed among the specimens, but specimens without bottom reinforcement and studs exhibited a rapid decrease in load capacity. In Figure 7a, the specimen with bottom tensile reinforcement, H5-T6-R-S-B, showed 74.4% higher displacement compared to the specimen without bottom tensile reinforcement, H5-T6-NR-S-B. This indicates that the bottom tensile reinforcement contributed to the distribution of the load, delayed crack initiation, and improved residual deformation capacity after yielding. The specimen with bottom studs, H5-T6-R-S-B, demonstrated 63.8% higher displacement compared to H5-T6-R-NS-B without studs. Moreover, the specimen with reinforced welding, H5-T6-R-NS-A, exhibited approximately 179.4% higher displacement compared to the specimen with basic welding, H5-T6-R-NS-B.

Similarly, as shown in Figure 7b, the specimen with bottom tensile reinforcement, H5-T8-R-S-B, exhibited a displacement of 2.21, which was significantly higher than that of the specimen without bottom tensile reinforcement, H5-T8-NR-S. The specimen without bottom studs, H5-T8-R-NS-B, showed a displacement of 1.01. Notably, the specimen with an 8 mm trapezoidal side reinforcement plate, H5-T8-R-S-B, demonstrated approximately 5.6% higher displacement compared to H5-T6-R-S-B. This suggests that as the thickness of the trapezoidal side reinforcement plate increases, both buckling resistance and stiffness improve, resulting in enhanced displacement capacity. Bottom tensile reinforcement significantly improved maximum displacement and ductility, ensuring stable behavior after yielding. Additionally, increasing the thickness of the trapezoidal side reinforcement plate enhanced buckling resistance and stiffness, thereby increasing displacement capacity. The welding method also improved the stiffness of the connection between the lipped channel and the trapezoidal side plate, contributing to greater displacement under maximum load conditions.

Stud anchors play a critical role in transferring shear forces between steel and concrete, maintaining composite action. However, the number of studs directly impacts construction costs and complexity. The vertical flanges of lipped channels can partially replace the role of stud bolts while maintaining composite action. Experimental results showed that even without studs (NS), the maximum load capacity of the composite beam decreased by only 7–9%. This demonstrates that reducing the number of studs can lower material costs and shorten construction periods. Additionally, it was confirmed that design requirements can be met using only basic welding without reinforced welding. Omitting reinforced welding reduces welding time and fabrication costs. Simplified welding methods facilitate easier quality control and contribute to faster construction speeds. Trapezoidal side reinforcement plates enhance stiffness, maintaining flexural and shear performance while reducing steel usage. Compared to conventional flat reinforcement plates, they provide the same buckling resistance and flexural strength while saving on steel consumption. By suppressing local buckling and increasing shear resistance area, it is possible to design structures that safely bear loads while minimizing steel use.

Therefore, designs utilizing lipped channels and trapezoidal side reinforcement plates not only reduce the complexity of structural components and improve durability, thereby lowering maintenance costs, but also contribute to the creation of cost-effective and environmentally sustainable structures.

### 3.2. Failure Modes

**Figure 8:**
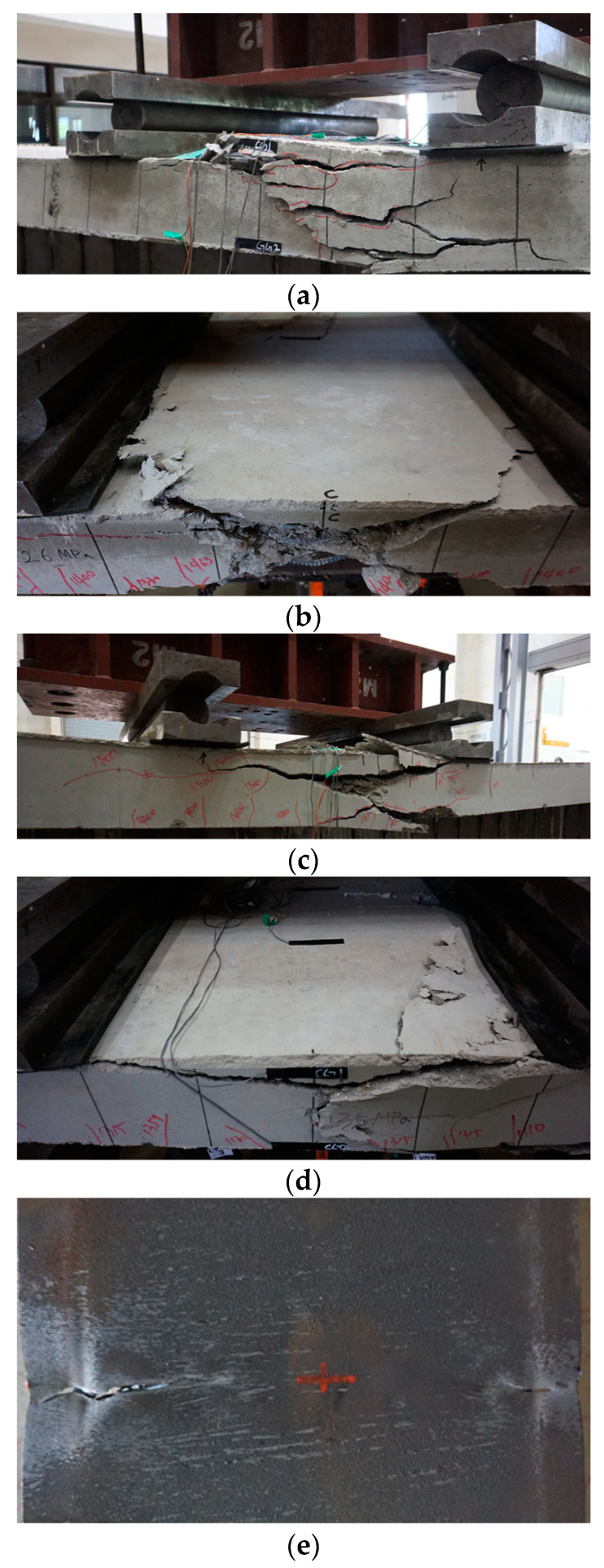
Failure shapes of concrete slab and bottom plate. (**a**) H5-T8-R-S-B; (**b**) H5-T8-R-NS-B; (**c**) H5-T8-R-S-A; (**d**) H5-T8-R-NS-A; (**e**) H5-T6-R-S-A.

Figure 8 shows the failure patterns of the concrete slab and steel parts of the specimens. The experiment was terminated when the load decreased to 80% of the maximum load. In most specimens, the load decreased due to the crushing of the concrete slab after reaching the maximum load. As depicted in Figure 8, flexural cracks appeared on the side of the concrete slab between the loading points. There was no significant buckling observed in the lipped channels. The load reduction was caused due to the concrete slab crushing after the maximum load was reached.

The H5-T6 series specimens, which have 6 mm-thick trapezoidal side plates, experienced a reduction in load not primarily due to the crushing of the concrete slab, as seen in Figure 8a–d, but rather due to a crack that first appeared at the welded joint in the central part of the trapezoidal side plate, as shown in Figure 8e. This crack subsequently affected the bottom steel plate, leading to its fracture and a subsequent reduction in load. The specimens with the presence or absence of bottom tensile reinforcement (R/NR) did not exhibit any buckling in the lipped channel. As shown in Table 6, the presence of bottom tensile reinforcement resulted in differences in experimental strength and maximum displacement (deflection). For the specimens with the presence or absence of bottom studs (S/NS), there were differences in deflection, but no significant impact on the test strength was observed. As shown in the results of Figure 8a, the presence or absence of bottom tensile reinforcement (R/NR) led to an increase in both strength and flexural deformation. Specimens without bottom tensile reinforcement exhibited relatively more cracks in the concrete slab, which likely affected the experimental strength and flexural deformation. The variation according to the welding reinforcement method (B/A) did not result in a significant difference in experimental strength, but there was a slight increase in the maximum displacement (deflection). In the H5-T6 series, all specimens except for the H5-T6-R-NS-A specimen experienced tensile fracture at the welded joint in the center of the trapezoidal side plate and the bottom plate. The H5-T6-R-NS-A specimen, however, had the experiment terminated due to the crushing of the concrete slab.

In the H5-T8 series, where the trapezoidal side reinforcement plates are 8 mm thick, concrete slab crushing occurred as shown in Figure 8. Similar to the H5-T6 series, tensile fracture occurred in the bottom plate. There was no slippage between the steel and concrete, nor did any buckling of the lip-shaped steel occur. The load–displacement curve in Figure 8b shows that there was no difference between the specimens with or without the bottom studs (S/NS) in the H5-T8 series. Regarding the presence or absence of bottom tensile reinforcement (R/NR), the progression of cracks in the concrete slab was less pronounced in specimens with bottom tensile reinforcement, leading to the conclusion that the presence of bottom tensile reinforcement impacts both the strength and flexural deformation. The H5-T8 series showed trends similar to the H5-T6 series when comparing different welding reinforcement methods (B/A). Among the H5-T8 series specimens, all except the H5-T8-NR-S-B specimen experienced concrete slab crushing and tensile fracture at the welded joint in the center of the trapezoidal side reinforcement plate. The H5-T8-NR-S-B specimen had the experiment terminated due to fracturing at the welded joint in the center of the trapezoidal side reinforcement plate and the bottom plate.

## 4. Comparison and Discussion of Flexural Behaviors

### 4.1. Strength Evaluation

The comparison between the experimental results and design predictions of the specimens is summarized in Table 6. The maximum bending moment of the composite beams with lipped channels was evaluated according to the American Institute of Steel Construction (AISC 360-22), the Korean Design Standards for Steel Structures in Buildings (KDS 41 30 10) [18], and the Design Standards for Composite Structures (KDS 14 31 80) [17]. For the calculation of the nominal flexural strength of composite members, both the plastic stress distribution method and the strain compatibility method are recommended [16,17,18,19]. In this study, the plastic stress distribution method was applied. The plastic neutral axis (P.N.A) was determined by evaluating the yield strength of the steel ($f_{y}$) and the compressive stress of the concrete (0.85$f_{ck}$). The tensile strength ($T_{s}$) of the steel is calculated by multiplying the steel cross-sectional area ($A_{s}$) by the yield strength ($f_{y}$). The compressive strength ($C_{c}$) of the concrete is calculated considering the effective width of the concrete slab ($b_{e}$) to determine the plastic neutral axis (P.N.A).

**Figure 9:**
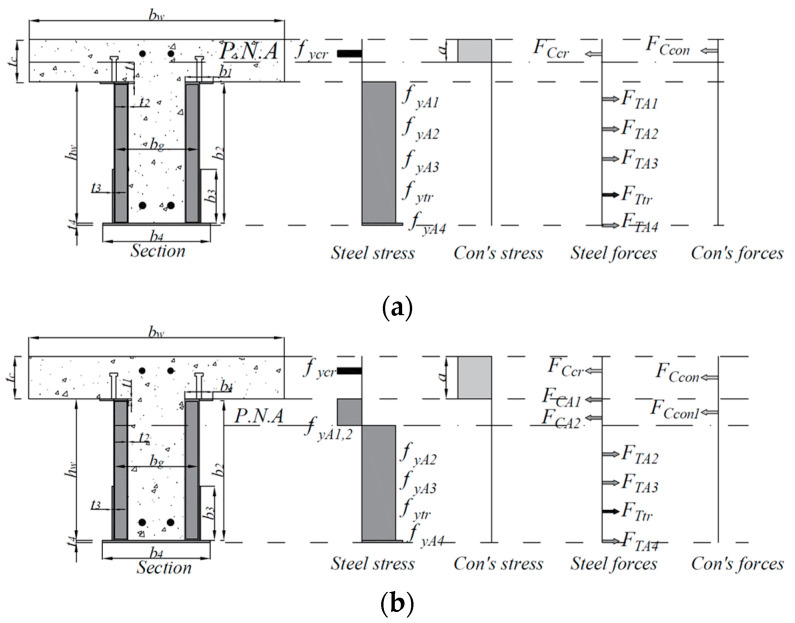
Stress distribution for calculation of positive plastic moment. (**a**) P.N.A in concrete slab; (**b**) P.N.A in steel beam.

The composite beam with lipped channels was evaluated using an existing method for calculating the nominal flexural strength of composite beams. Since the steel beam includes bottom reinforcement, if the plastic neutral axis (P.N.A) is located within the concrete slab as shown in Figure 9a, the nominal flexural strength ($M_{n}$) of the composite beam is defined by Equation (1), and the plastic neutral axis (P.N.A) is defined by Equation (3). Additionally, if the plastic neutral axis (P.N.A) is located within the steel beam, as shown in Figure 9b, the nominal flexural strength of the composite beam is defined by Equation (2), and the plastic neutral axis (P.N.A) is defined by Equation (4). The flexural strength of the composite beam with lipped channels is calculated by multiplying the tensile force of each component (lipped channel, trapezoidal side plate, and bottom plate) by the distance between the center of the concrete compressive force and the center of each tensile force. The test specimens were designed based on the assumption of a fully composite beam, ensuring that the plastic neutral axis (P.N.A) would be located within the concrete slab.

(1) $$M_{n}=F_{TA1}jd_{TA1}+F_{TA2}jd_{TA2}+F_{TA3}jd_{TA3}+F_{TA3}jd_{TA4}+F_{TA4}jd_{TA4}+F_{Ttr}jd_{Ttr}$$

(2) $$M_{n}=F_{TA2}jd_{TA2}+F_{TA3}jd_{TA3}+F_{TA4}jd_{TA4}+F_{Ttr}jd_{Ttr}+F_{Ccon}jd_{Ccon}+\;F_{Ccon1}jd_{Ccon1}+F_{CA1}jd_{CA1}+F_{CA2}jd_{CA2}$$

(3) $$P.N.A=\frac{A_{s1}f_{y1}+A_{s2}f_{y2}+A_{s3}f_{y3}+A_{s4}f_{y4}+A_{sty}f_{ytr}-A_{scr}f_{ycr}}{0.85f_{ck}b_{w}}\;$$

(4) $$P.N.A\;=\frac{2f_{y2}t_{2}\left(2t_{c}+b_{w}\right)+2f_{y3}b_{3}t_{3}+f_{y4}b_{4}t_{4}-0.85f_{ck}t_{c}\left(b_{w}-b_{g}\right)+A_{tr}f_{tr}-A_{cr}f_{cr}}{0.85f_{ck}b_{w}+4f_{y2}t_{2}}$$

Here, $A_{1}$ = top sub-platform, $A_{2}$ = lipped channels, $A_{3}$ = side plates, $A_{4}$ = bottom plates, $A_{tr}$ = tension reinforcement, $A_{cr}$ = compression reinforcement, $A_{s1}$ = top sub-platform area ($mm^{2}$), $A_{s2}$ = lipped channels area ($mm^{2}$), $A_{s3}$ = side plates area ($mm^{2}$), $A_{s4}$ = bottom plates area ($mm^{2}$), $A_{str}$ = tension reinforcement area ($mm^{2}$), $A_{scr}$ = compression reinforcement area ($mm^{2}$), $f_{y1}$ = top sub-platform yield strength (MPa), $f_{y2}$ = lipped channels yield strength (MPa), $f_{y3}$ = side plates yield strength (MPa), $f_{y4}$ = bottom plates yield strength (MPa), $f_{ytr}$ = tension reinforcement yield strength (MPa), and $f_{ycr}$ = compression reinforcement yield strength (MPa).

The calculated flexural strength ($M_{n}$), based on the material test results of the specimens, and the maximum bending moment ($M_{e}$) from the experimental results are summarized. The maximum bending moment from the experimental results was calculated as the bending moment at the maximum load, and the nominal flexural strength was determined by changing the position of the plastic neutral axis (P.N.A.) for each specimen. When applying the material test results of concrete and steel, it was found that the plastic neutral axis (P.N.A.) for most specimens was located within the concrete slab. In experiments where the trapezoidal side plate was welded at the center, stress concentration near the weld joint of the central part of the trapezoidal side plate affected the flexural strength of the specimens. For composite beams without bottom tensile reinforcement, such as specimens H5-T6-NR-S-B and H5-T8-NR-S-B, the maximum flexural strength was up to 11% lower than the theoretical flexural strength without applying the strength reduction factor due to early tensile fracture of the bottom plate by tensile fracturing at the welded joint in the central part of the trapezoidal side plate. However, considering the flexural strength reduction factor of $\phi_{b}$ = 0.9, the average flexural strength ratio ($M_{e}/\phi_{b}M_{n}$) is 1.08, and the maximum experimental flexural strength can be considered safe.

In conventional composite beams, stud anchors play a critical role in transferring shear forces between the steel element and concrete slab, preventing slip and maintaining composite action. In this study, it was confirmed that the vertical flange of the lipped channel partially replaces the role of studs, acting as a shear connector between the steel and concrete. The vertical flange, being in direct contact with the slab, transfers shear forces and reduces slip, thereby maintaining composite action. When the thickness of the trapezoidal side plates increased from 6 mm to 8 mm, the flexural strength of the composite beam improved. The trapezoidal side plates enhance both flexural and shear strength in composite beams by suppressing buckling and increasing sectional stiffness. As the thickness of the trapezoidal side plates increases, the moment of inertia of the section also increases, thereby improving flexural performance. Additionally, the inclusion of trapezoidal side plates in the strength equation increases the flexural capacity, and the increase in thickness results in a greater shear resistance area. In the absence of bottom tensile reinforcement, the trapezoidal side reinforcement plates play a key role in resisting tensile forces, significantly influencing the flexural capacity [25].

### 4.2. Strain Distribution

**Figure 10:**
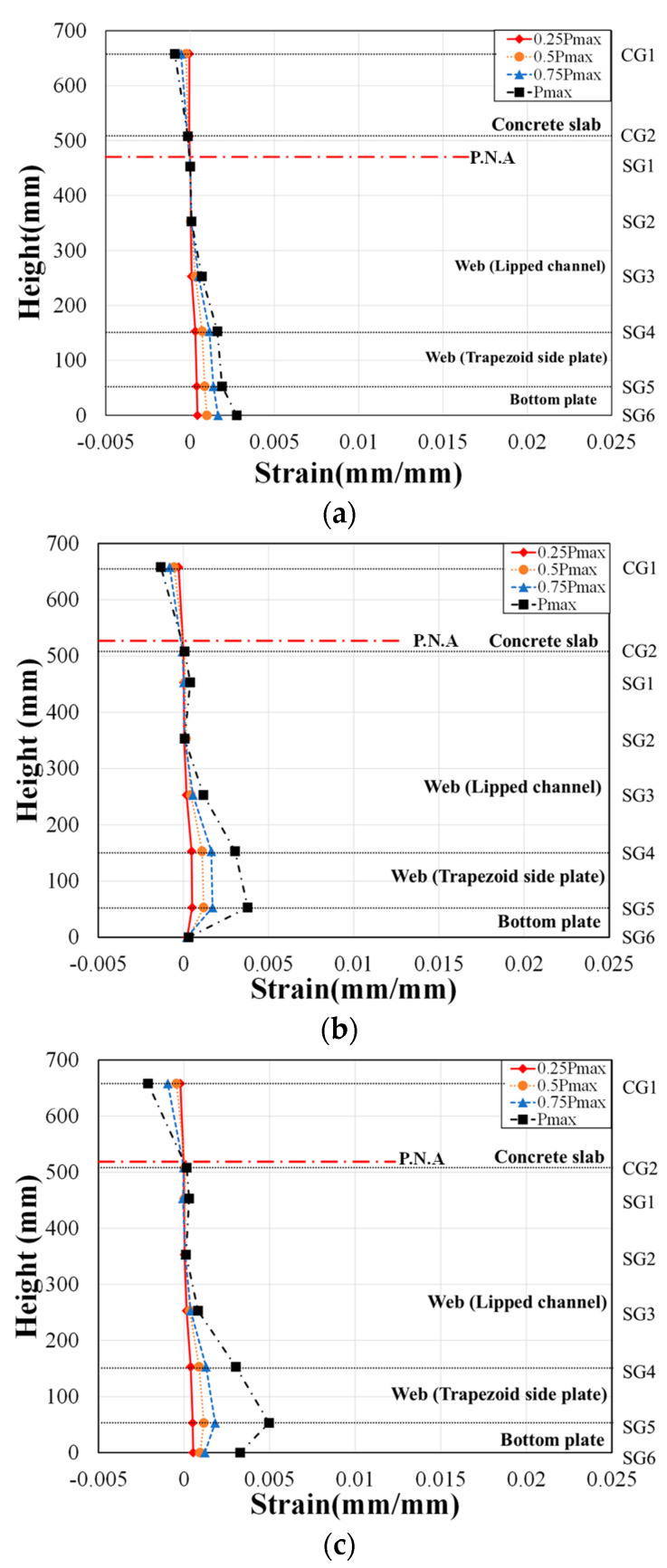

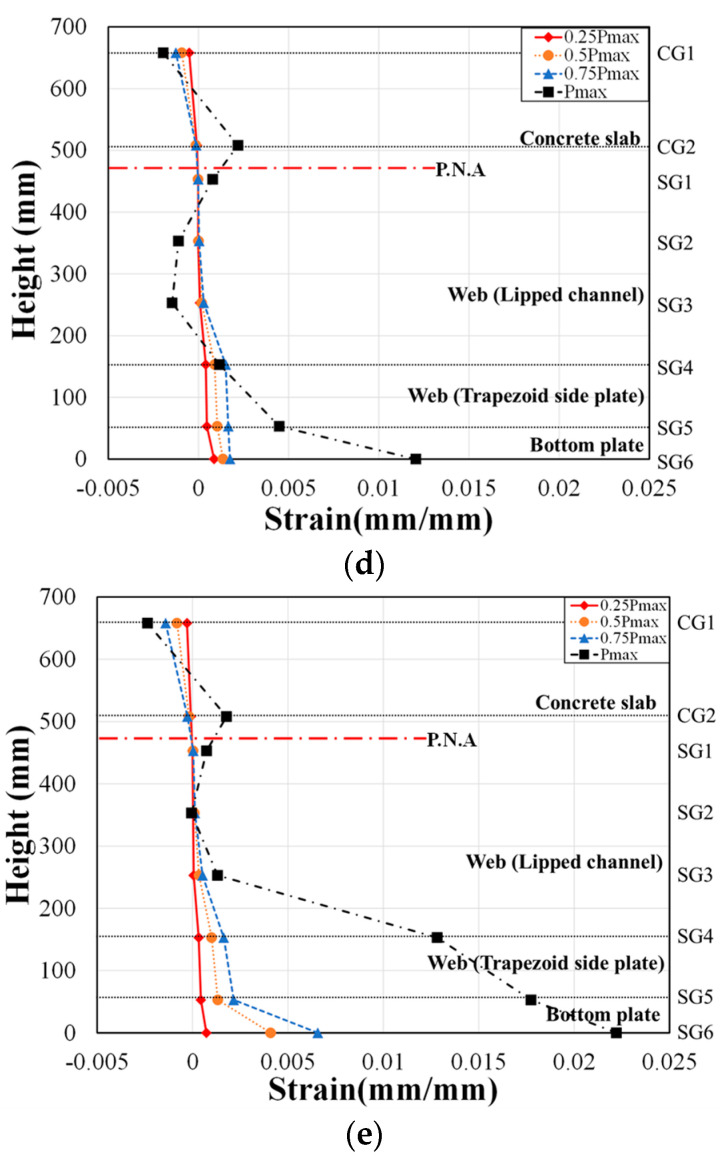
Strain distribution of H5-T6 series. (**a**) H5-T6-R-S-B; (**b**) H5-T6-NR-S-B; (**c**) H5-T6-R-NS-B; (**d**) H5-T6-R-S-A.

**Figure 11:**
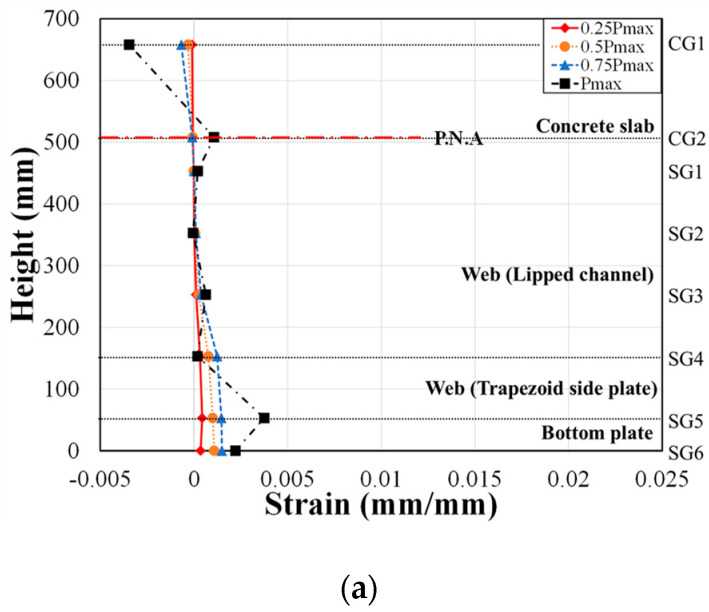

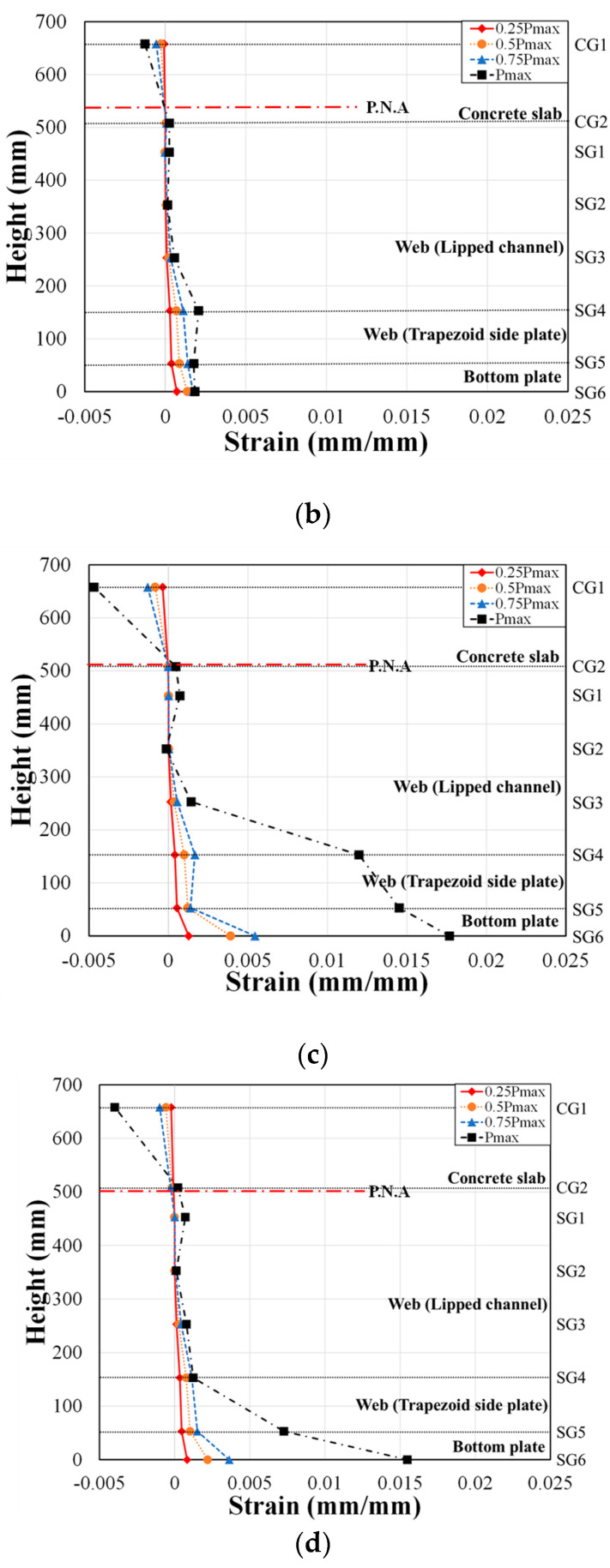

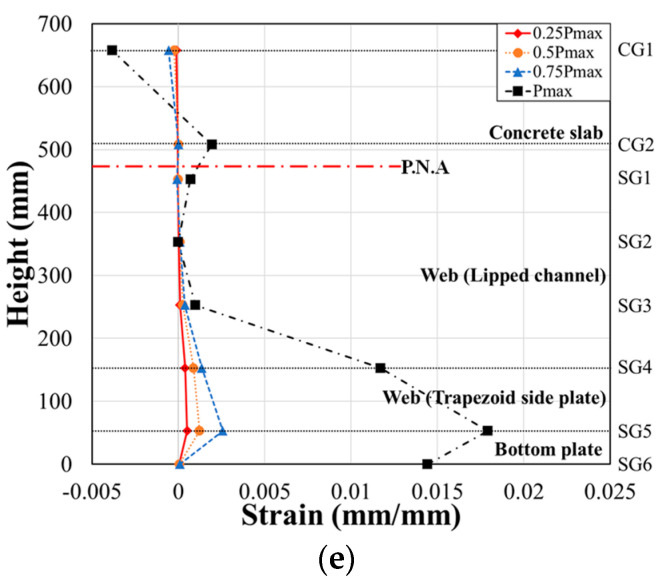
Strain distribution of H5-T8 series. (**a**) H5-T8-R-S-B; (**b**) H5-T8-NR-S-B; (**c**) H5-T8-R-NS-B; (**d**) H5-T8-R-S-A; (**e**) H5-T8-R-NS-A.

To assess the strain behavior of the composite beam with lipped channels, strain gauges were attached at various locations, as shown in Figure 4. These locations include the concrete slab (CG1, CG2), steel web (lipped channel SG1, SG2, SG3), trapezoidal side plates (SG4, SG5), and bottom plate (SG6). Strain was measured at these points to monitor the structural response at different load levels. The height–strain measurements at the bottom plate were recorded at 25%, 50%, 75%, and 100% of the maximum load and are summarized in Figure 10 and Figure 11.

In Figure 10 and Figure 11, it is observed that the tensile strains measured by strain gauges attached to the steel web of the composite beam increased up to 75% of the maximum load. Under this load, no strains exceeding the yield strains of the material specimens were observed. The yield strains for the materials are as follows: lipped channel (3.2 mm) = 0.003373, trapezoidal side plates (6.0 mm) = 0.004495, (8.0 mm) = 0.002115, and bottom plate (8.0 mm) = 0.005355.

Figure 10 illustrates the strain distribution at the four stages of the H5-T6 series specimens. Based on the material test results summarized in Table 6, the calculated plastic neutral axis (P.N.A) was located within the concrete slab. However, as shown in Figure 10, the actual neutral axis observed from the strain gauges was positioned within the beam, close to the bottom of the slab, for all specimens except for H5-T6-NR-S-B and H5-T6-R-NS-B. The strain in the lipped channel (SG1) was nearly zero but increased when it entered the tensile region at the maximum load. As seen in Figure 10b,c, the strains in the lipped channel for specimens H5-T6-NR-S-B and H5-T6-R-NS-B decreased at 75% of the maximum load.

Figure 11 shows the strain distribution for the H5-T8 series specimens. Although the calculated plastic neutral axis (P.N.A) based on material test results, as presented in Table 6, was within the concrete slab, the actual neutral axis observed from the strain gauges in Figure 11 was located inside the beam for specimens H5-T8-R-S-A and H5-T8-R-NS-A. For the H5-T8 series, the strains in the lipped channel (SG2) and the side plate reinforcement (SG4) in specimen H5-T8-R-S-B decreased at 75% of the maximum load.

The plastic neutral axis, as calculated from material test results, was found to be within the concrete slab, consistent with the design. However, the neutral axis predicted from the strain gauges was observed to be inside the beam, closer to the bottom of the slab in some specimens. The neutral axis measured by the strain gauges was 6.9% to 40.8% higher than that calculated from the material tests.

## 5. Conclusions

From the flexural experiments on U-shaped composite beams with lipped channels, the following conclusions were drawn:

(1) All specimens of the composite beam with lipped channels exhibited flexural cracks in the concrete slab as the load increased. However, it was observed that the stress concentration near the weld joint at the center of the trapezoidal side reinforcement plate, which was welded in the middle, influenced both the fracture mode and the experimental flexural strength.

(2) Specimens with bottom tensile reinforcement (R) demonstrated an average increase of approximately 30% in maximum strength compared to specimens without bottom reinforcement (NR). This indicates that bottom tensile reinforcement makes a significant contribution to the flexural behavior and maximum load capacity of composite beams.

(3) Specimens with bottom studs (S) showed an average increase of 8% in maximum strength. The vertical flanges of the lipped channels, which make up the composite beam web, enabled composite action with the concrete, potentially replacing the role of stud bolts and contributing to integrated behavior.

(4) When reinforced welding (A) was applied, the maximum strength slightly increased or remained similar compared to basic welding (B). The welding method (B/A) had a minimal impact on structural performance.

(5) The evaluation of experimental strength using the plastic stress distribution method revealed that the experimental flexural strength of certain specimens without tensile reinforcement (H5-T6-NR-S-B, H5-T8-NR-S-B) was lower than the nominal flexural strength. This indicates that stress concentration at the weld joint in the center of the side reinforcement plate negatively affected experimental strength. However, the average ratio of experimental flexural strength to the design flexural strength, considering the strength reduction factor ($\phi_{b}$), was 1.08, confirming that the specimens exhibited safe behavior within the design standards.

In the future, based on the experimental results of the materials and composite beams, a finite element analysis model will be developed to perform parametric analysis on variables such as the dimensions of the lipped channel section, the thickness and height of the side trapezoidal plates, the thickness of the bottom plates, and the steel grade. It is necessary to verify the design equations and to conduct a detailed analysis of the flexural behavior of the developed U-shaped composite beam.

## Figures and Tables

**Table 1:** Concrete mix proportion.

Design Criterion Strength *f_ck_* (MPa)	W/B (%)	S/A (%)	Unit Amount of Material $(kg/m^{3}$)				
24	49.4	49.4	W	C	G	S	AE
			179	254	889	858	2.53

Note: W/B = water/binder ratio; S/A = fine aggregate ratio; W = water; C = cement; G = coarse aggregate; S = fine aggregate; AE = water-reducing admixture.

**Table 2:** Tensile test results of stud.

Coupon	*D_e_* (mm)	$E_{s}$ (GPa)	$f_{y}$ (MPa)	$f_{u}\;\;$ (MPa)	$\varepsilon_{elo}\;$ (%)
∅16(HS1)	12.11	186.7	309.8	421.3	33.1
∅19(HS1)	11.90	179.5	303.3	421.3	23.9

Note: $D_{e}$ = diameter of tensile coupon; $E_{s}$ = elastic modulus; $f_{y}$ = yield stress; $f_{u}$ = tensile strength; $\varepsilon_{elo}$ = elongation at fracture.

**Table 3:** Tensile test results of rebar.

Coupon	$E_{s}\;$ (GPa)	$f_{y}\;$ (MPa)	$f_{u}\;$ (MPa)	$\varepsilon_{elo}\;$ (%)
D10(SD400)	187.8	421.4	554.5	23.2
D22(SD400)	188.3	475.3	629.3	16.8
D25(SD500)	190.1	564.8	710.7	21.4

**Table 4:** Tensile test results of steel.

Coupon	$t_{e}\;$ (mm)	$E_{s}\;$ (GPa)	$f_{y}\;$ (MPa)	$f_{u}\;$ (MPa)	$\varepsilon_{elo}$ (%)
3.2 mm (SS275)	3.15	184.5	267.1	418.1	32.0
4.0 mm (SS275)	3.95	187.4	322.3	454.5	29.5
6.0 mm (SM355A)	6.04	186.1	429.9	569.8	26.4
8.0 mm (SS275)	7.71	186.8	314.0	469.9	37.6
8.0 mm (SM355A)	7.82	187.2	384.5	533.3	33.4

**Table 5:** Test results.

Specimens	$P_{n}$ (kN)	$P_{ue}$ (kN)	$P_{y}$ (kN)	$P_{ue}/P_{n}$	$P_{ue}/P_{y}$	$\delta_{ue}$ (mm)	$\delta_{y}$ (mm)	$\delta_{ue75}$ (mm)
H5-T6-R-S-B	1448.4	1464.3	1108.3	1.01	1.32	62.1	16.97	20.6
H5-T6-NR-S-B	1234.8	1094.9	884.1	0.89	1.24	35.6	15.06	18.1
H5-T6-R-NS-B	1448.4	1365.7	1123.2	0.94	1.22	37.9	17.71	19.4
H5-T8-R-S-B	1436.4	1406.2	1052.8	0.98	1.34	65.6	16.12	19.1
H5-T8-NR-S-B	1222.2	1091.4	895.3	0.89	1.22	29.6	13.64	16.1
H5-T8-R-NS-B	1436.4	1469.2	1096.5	1.02	1.34	66.9	15.99	19.5
H5-T6-R-S-A	1448.4	1406.3	1058.6	0.97	1.33	67.5	17.45	20.2
H5-T6-R-NS-A	1448.4	1498.4	1113.7	1.03	1.35	82.6	18.97	23.2
H5-T8-R-S-A	1436.4	1507.7	1117.1	1.05	1.35	76.5	17.03	20.9
H5-T8-R-NS-A	1436.4	1359.1	1074.5	0.95	1.26	68.1	16.63	19.6

**Table 6:** Comparison of design predictions and test results.

Specimens	$P_{ue}$ (kN)	$M_{e}$ (kN·m)	$M_{n}\;$ (kN·m)	$\phi_{b}M_{n}\;$ (kN·m)	$M_{e}/M_{n}$	$M_{e}/\phi_{b}M_{n}$	$\delta_{ue}$ (mm)	P.N.A (mm)
H5-T6-R-S-B	1461.3	1717.0	1701.9	1531.7	1.01	1.12	62.1	138.5
H5-T6-NR-S-B	1094.9	1286.5	1450.9	1305.8	0.89	0.99	35.6	116.8
H5-T6-R-NS-B	1365.7	1604.7	1701.9	1531.7	0.94	1.05	37.9	138.5
H5-T8-R-S-B	1406.2	1652.3	1687.8	1519.0	0.98	1.09	65.6	137.2
H5-T8-NR-S-B	1091.4	1282.4	1436.0	1292.4	0.89	0.99	29.6	115.4
H5-T8-R-NS-B	1469.2	1726.3	1687.8	1519.0	1.02	1.14	66.9	137.2
H5-T6-R-S-A	1406.3	1652.4	1701.9	1531.7	0.97	1.08	67.5	138.5
H5-T6-R-NS-A	1498.4	1760.7	1701.9	1531.7	1.03	1.15	82.6	138.5
H5-T8-R-S-A	1507.7	1771.6	1687.8	1519.0	1.05	1.17	76.5	137.2
H5-T8-R-NS-A	1359.1	1596.9	1687.8	1519.0	0.95	1.05	68.1	137.2
Average	-	-	-	-	0.97	1.08	-	-

$P_{ue}$ = test maximum load; $M_{e}$ = test flexural strength; $M_{n}$ = nominal flexural strength based on the material test result; $\phi_{B}M_{n}$ = design flexural strength; $\delta_{ue}$ = displacement of the specimen under the test maximum load; P.N.A = values calculated from material test results.

## Data Availability

The data that support the findings are available from the corresponding author upon request.
